# A functional missense variant in *MRI1* links methionine salvage to reduced type 2 diabetes risk

**DOI:** 10.1016/j.isci.2026.117243

**Published:** 2026-08-20

**Authors:** Khushdeep Bandesh, Michael Traurig, Kendrick Freeland, Paolo Piaggi, Leslie J. Baier

**Affiliations:** 1 Phoenix Epidemiology and Clinical Research Branch, National Institute of Diabetes and Digestive and Kidney Diseases, National Institutes of Health, Phoenix, AZ, USA

**Keywords:** T2D genetics, methionine metabolism, methylthioribose-1-phosphate isomerase, MTNA, MRDI, stability-activity tradeoff

## Abstract

Type 2 diabetes (T2D) pathogenesis is shaped by both risk and protective genetic variants, but mechanisms underlying disease protection remain understudied. Previously, in a population isolate with high T2D prevalence, we unexpectedly identified a relatively frequent protective missense variant (Arg149Cys, rs551664067 C/T) in the methionine salvage gene *MRI1*, never reported for T2D. MRI1 is expressed in human pancreatic islets and insulin-responsive tissues, with islet expression correlating with insulin expression. Using MRI1’s resolved crystal structure, we show Arg149Cys substitution compacts the ligand-binding pocket by >53%, and enzyme kinetics confirm enhanced substrate engagement and 18.6% improved catalytic flux, boosting methionine salvage for downstream pathway enzymes. Circulating methionine levels inversely correlated with insulin sensitivity, consistent with enhanced MRI1 activity channeling methionine into salvage rather than pathological accumulation. Together, MRI1 is identified as a metabolic regulator that links enzyme biophysics to efficient methionine recycling and T2D protection, showcasing quantitative NMR as a genetics-to-mechanism bridge.

## Highlights

• MRI1 Arg149Cys protective gain-of-function variant delays T2D onset by 5 years
• MRI1 Cys149 shows stability-activity trade-off with enhanced catalytic kinetics
• Arg149Cys enables efficient methionine salvage conferring T2D protection
• High-resolution ^1^H-NMR spectra of 3 key methionine salvage intermediates provided

## Graphical abstract

**Figure:**
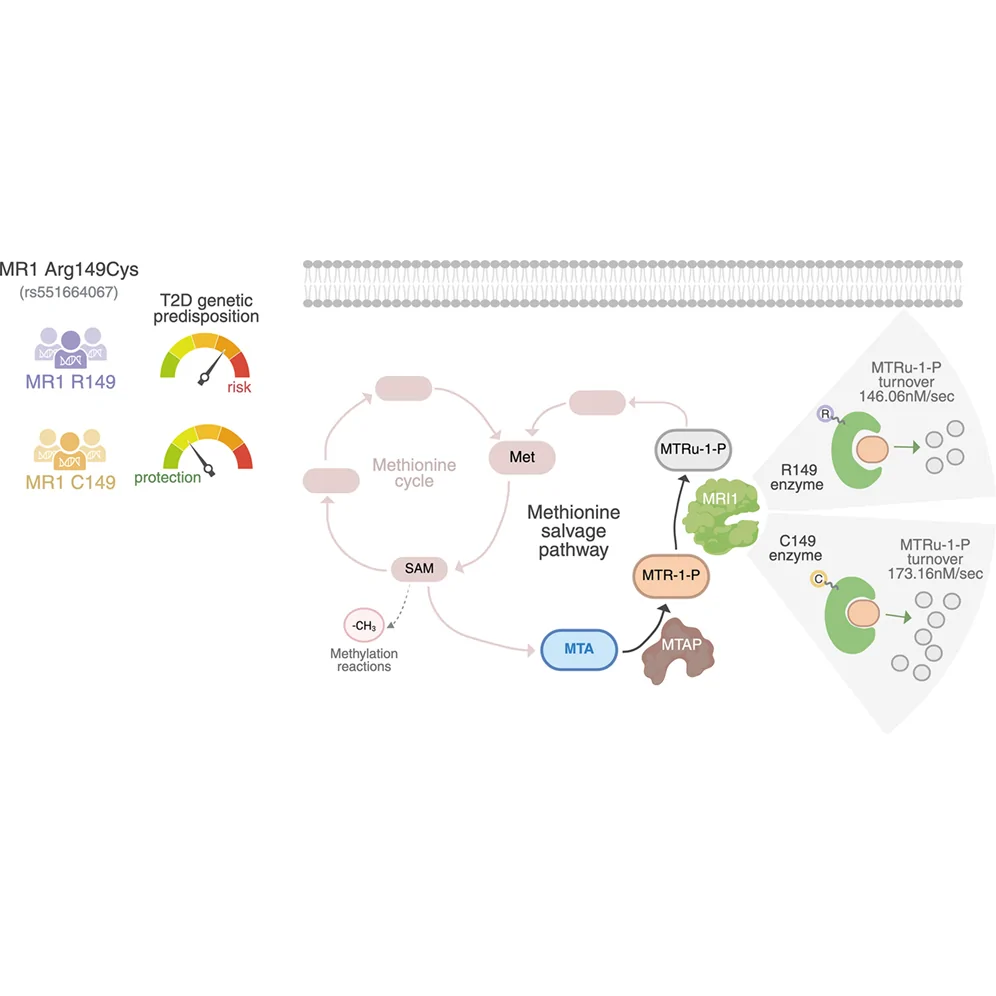

## Teaser

Spectroscopy; Molecular biology; Data analysis in structural biology

## Introduction

Type 2 diabetes (T2D) is characterized by complex interactions between insulin resistance and progressive pancreatic islet dysfunction. While genetic studies have identified 611 loci associated with T2D risk,^1^ functional insights into how coding variants influence disease susceptibility have been understood for only a handful of variants,^2^^,^^3^^,^^4^^,^^5^^,^^6^^,^^7^^,^^8^ with protective variants remaining especially understudied.^9^

We previously conducted a comprehensive analysis of coding variants associated with T2D^10^ in a population isolate with a high prevalence of T2D,^11^ identifying significant gene-based associations driven by missense variants in established diabetes genes – *ABCC8* (odds ratio [OR] = 2.2, *p* = 9.3 × 10^−6^, primary variant Arg1420His)^10^ and *MC4R* (OR = 2.6, *p* = 1.2 × 10^−5^, primary variants Gly34fs, Arg165Gly, and Arg165Gln).^10^ However, we also found a missense variant in an uncharacterized gene, *MRI1* (Arg149Cys, rs551664067 C/T) that exhibited a comparable but protective association with T2D (OR = 0.49 [0.36–0.66], *p* = 4.3 × 10^−6^).^10^ Despite having a protective effect, the MRI1 Arg149Cys substitution was highly enriched in this population isolate, where the protective Cys (T) allele had a frequency of 0.01 as compared with broader population cohorts such as TOPMED (0.000019) and ExAC (0.000017).^12^ *MRI1* encodes a crucial methionine salvage enzyme (methylthioribose-1-phosphate isomerase)^13^ and has never been implicated in T2D. Carriers of the Cys149 (T) allele exhibited a lower prevalence of T2D and a significantly delayed age of disease onset (51.2 in carriers vs. 46.2 years in non-carriers, *p* = 1.6 × 10^−5^). Given the previously unappreciated role of MRI1 in T2D, these compelling genetic associations prompted further investigation of the identified missense variant.

Here, we integrated human genetics with structural, biophysical, and enzymatic analyses to define the functional impact of the MRI1 Arg149Cys variant. We show that the substitution preserves the global protein structure but enhances the ligand-binding capacity and catalytic flux of MRI1 enzyme, offering a plausible mechanistic basis for its protective association with T2D.

## Results

### Stable MRI1 expression in T2D suggests enzyme activity as the driver of variant effects

**Figure 1:**
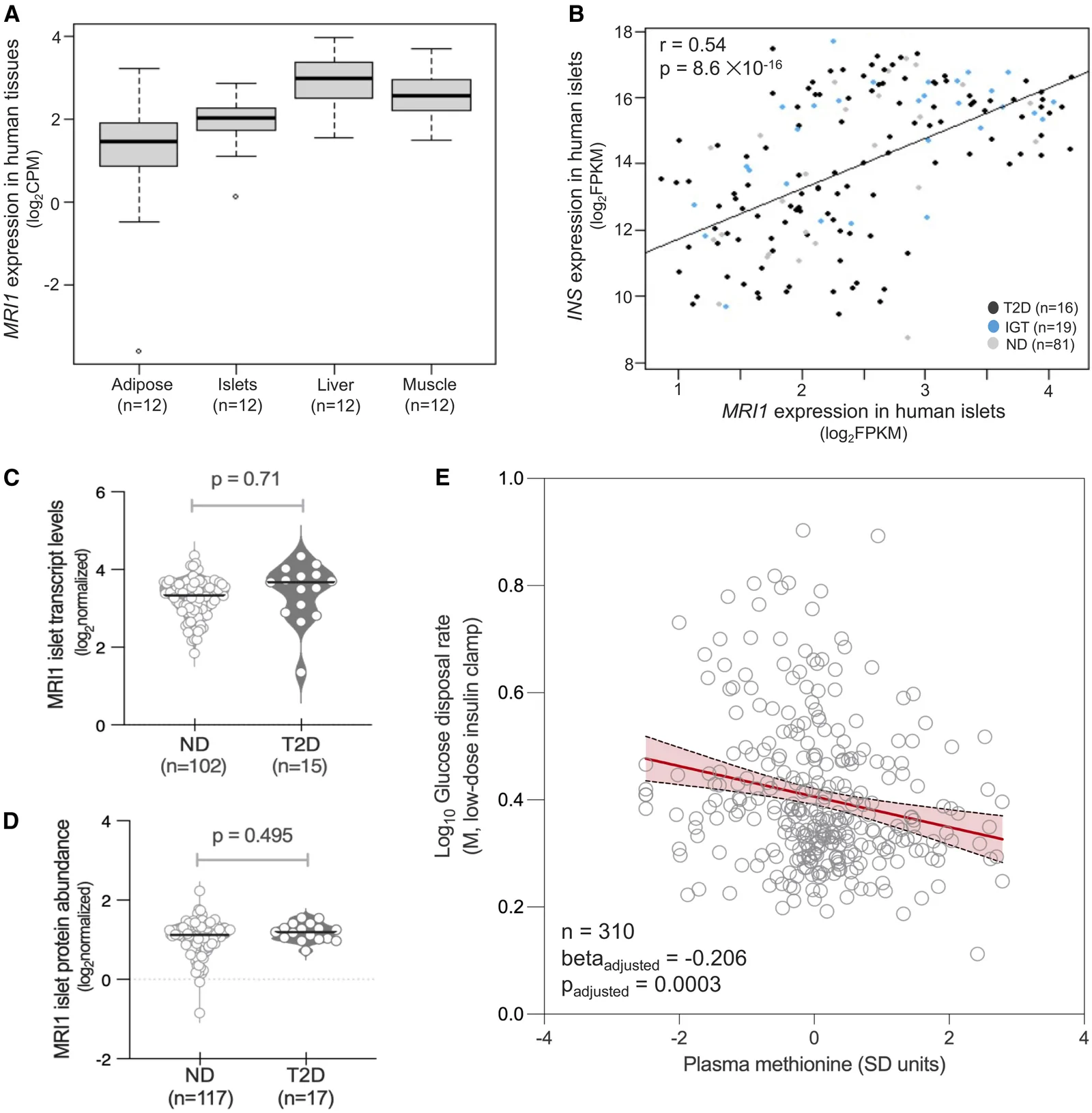
MRI1 expression across human tissues and its correlation with systemic insulin sensitivity (A) Boxplots showing *MRI1* gene expression in human pancreatic islets and major insulin-responsive tissues, all from the same 12 donors. Expression levels are shown as log_2_ counts per million (log_2_CPM) as reported in Islet Gene View,^14^ and the black solid lines denote median gene expression; *n* indicates the number of samples analyzed per tissue type from human donors. (B–D) Correlation between islet *MRI1* expression and *INS* expression, presented as log_2_ fragments per kilobase of transcript per million mapped reads (log_2_ FPKM). Each data point is an individual donor sample, colored by clinical group: type 2 diabetes (T2D, HbA1c > 6.5%), impaired glucose tolerance (IGT, HbA1c = 6%–6.5%), and non-diabetic (ND, HbA1c < 6.0%); *n* denotes number of human islet donors per clinical group. The solid line represents the linear regression fit. Spearman’s correlation coefficient (r) and *p* value are indicated. MRI1 transcript (C) and protein (D) levels in ND vs. T2D islets in HumanIslets.com cohort.^15^*n* indicates the number of human islet donors investigated per clinical group. The black solid lines denote median expression (median log_2_normalized MRI1 expression [interquartile range]: ND: RNA = 3.34 [0.61], protein = 1.13 [0.42]; T2D: RNA = 3.67 [0.85], protein = 1.19 [0.26]). (E) Correlation between plasma methionine levels (shown as standard deviation [SD] units from the population mean) and glucose disposal rate measured during a low-dose insulin clamp, expressed as log_10_-transformed M_low_ values. Each circle represents an individual; *n* indicates number of individuals investigated. Raw, unadjusted data are shown. The red line indicates the linear regression fit with the shaded area representing the 95% confidence interval. Spearman’s r and, age- and sex-adjusted *p* value are shown.^16^

We interrogated publicly available human gene expression datasets and found that *MRI1* is expressed at comparable levels in human pancreatic islets and major insulin-responsive tissues collected from the same 12 donors (Islet Gene View,^14^ Figure 1A). Within islets, *MRI1* is expressed at biologically relevant levels; its expression ranks at the 36^th^ percentile among expressed genes, with transcript abundance similar to that of *KCNJ11* (Figure S1)^14^ and positively correlated with insulin (*INS*) expression (r = 0.54, *p* = 8.6 × 10^−16^, Figure 1B),^14^ both of which are functionally critical islet genes. Islets from human donors without diabetes (ND) and those with diabetes (T2D) do not differ in *MRI1* expression at either the RNA (HumanIslets.com,^15^ adjusted *p* = 0.71, Figure 1C) or the protein levels (HumanIslets.com,^15^ adjusted *p* = 0.495, Figure 1D). Our group previously investigated circulating metabolites in individuals from the same population isolate where the *MRI1* variant was detected.^16^ Consistent with MRI1’s role in restoring methionine in the body, we found a modest inverse correlation between plasma methionine levels and insulin sensitivity (M_low_, age- and sex-adjusted beta = −0.206, *p* = 0.0003, Figure 1E).^16^ Together, these data led us to speculate that *MRI1*-related metabolic effects might be mediated by changes in protein (enzyme) activity rather than an altered gene or protein abundance.

### Conserved Arg149Cys substitution preserves MRI1 structure but reduces protein stability

**Figure 2:**
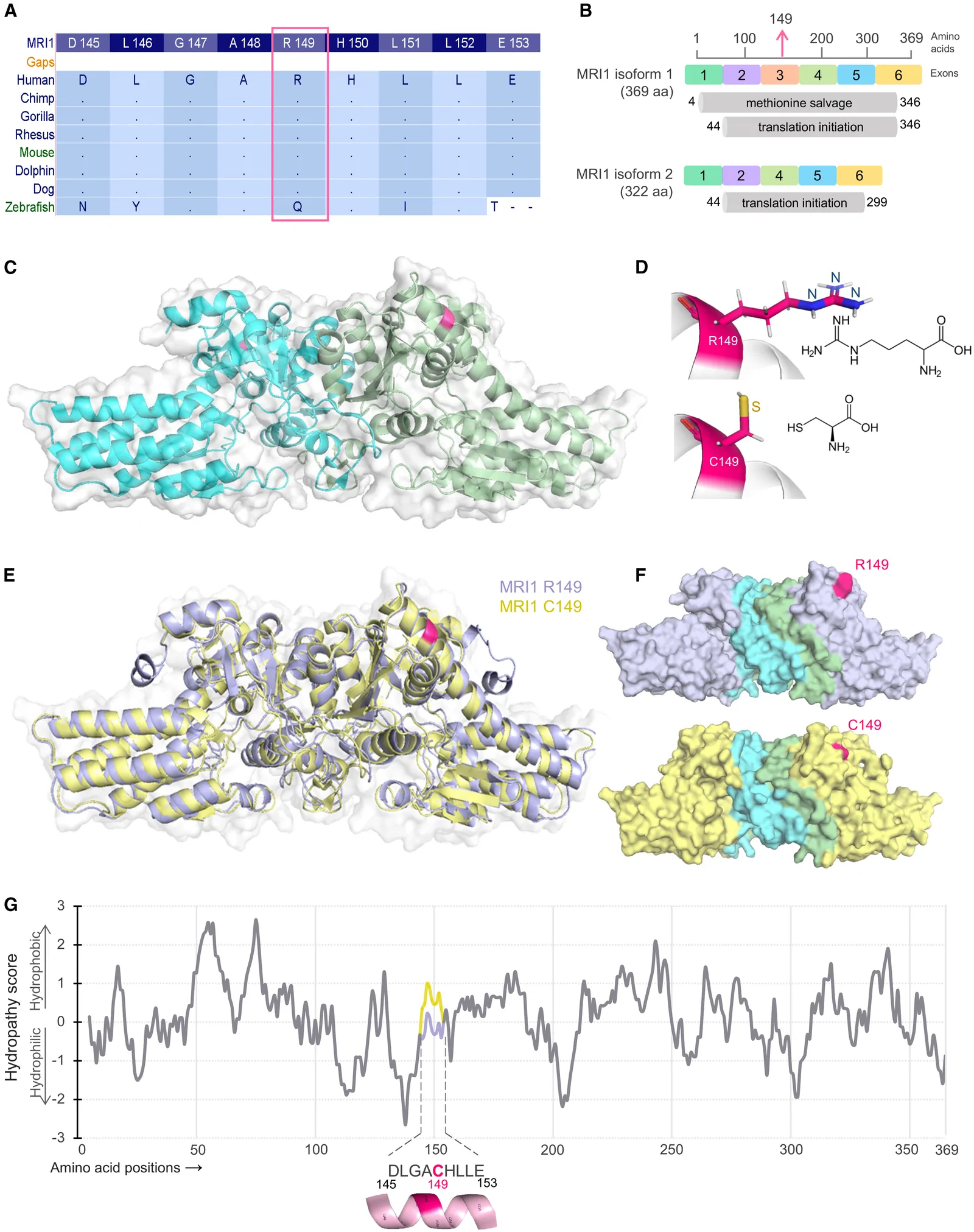
Sequence context of evolutionary conserved MRI1 Arg149Cys substitution (A) Multiple sequence alignment of the MRI1 protein across vertebrate species showing high evolutionary conservation (UCSC genome browser).^17^ Amino acid positions are indicated above the alignment. Human residues are shown explicitly, with dots indicating identity and non-identical residues indicated by substituted amino acids. The arginine residue at position 149 (R149) is highlighted (magenta box), indicating a highly conserved site across species. (B) Schematic representation of conserved protein domains^18^ in MRI1 isoforms—full-length (369 amino acids) and short isoform (322 amino acids). Presence of exon 3 which harbors R149 is critical for the presence of the methionine salvage domain. (C) Available X-ray crystal structure of the human MRI1 protein resolved at 2.50 Å (PDB ID: 4LDQ) is shown in ribbon representation (showing secondary structures) over a molecular surface model rendered in light gray. Monomers of MRI1 homodimer are colored cyan and light green, respectively. Residue R149, which resides within a long α-helical region, is highlighted in magenta. (D) Stick representation of residue R149 and its spatial comparison with C149. (E) A 3D superimposition of the two MRI1 proteins (Arg149, lavender colored and Cys149, yellow colored) shows structural similarity. Root means square deviation (RMSD, generated by PyMol) between the two structures is 1.77 Å. (F) Residue 149 is spatially distant from the dimerization interface between the two MRI1 monomers, which are shown in cyan and light green corresponding to each monomer. (G) Kyte-Doolittle Hydropathy plots of the two MRI1 proteins across amino acid positions, generated by ProtScale (Expasy) with the default window size. Higher score indicates higher hydrophobicity. Region spanning residue 149 and residues in adjacent upper and lower α-helical turns, exhibit increased hydrophobicity following the Arg149Cys substitution.

Cross-species sequence alignment showed that Arg149 is evolutionarily conserved (Figure 2A) and its substitution to Cys is predicted to be poorly tolerated (SIFT score = 0.05 and PolyPhen2 score = 1.0). Analysis of conserved domains revealed that exon 3, which harbors the Arg149Cys substitution, is essential for salvage activity; indeed, the short MRI1 isoform (322 amino acids) missing exon 3 also lacks the methionine salvage domain (Figure 2B), underscoring the importance of this region.

MRI1 functions as a homodimer, and residue 149 is in a long α-helix that connects the N-terminal and C-terminal domains within each monomer (Figure 2C). Arginine is a positively charged amino acid, while cysteine is a small, neutral thiol-containing amino acid (Figure 2D). This shift in charge can potentially alter a protein’s structural stability, local or global electrostatics, and molecular interactions.

A complete X-ray crystal structure of native MRI1 (Arg149) protein has been previously determined at a 2.50 Å resolution (PDB ID: 4LDQ); which allowed us to interrogate the molecular consequences of Arg149Cys substitution. Using this structure as a template and homology-based modeling, we generated the 3D structure of MRI1 Cys149 protein and compared its structural properties with the native protein. 3D superimposition of the Arg149 and Cys149 protein homodimers indicated preserved overall folding with a root-mean-square deviation (RMSD) of 1.77 Å ([369 residues × 2]_Arg149_ × [369 residues × 2]_Cys149_ comparisons, Figure 2E). The substitution did not introduce unfavorable backbone conformations, as both residues occupy favored regions of φ/ψ dihedral space. Structural mapping further showed that residue 149 is spatially distant from the monomer-monomer interaction interface (Figure 2F), indicating that the Arg149Cys substitution is unlikely to affect MRI1 dimerization. Because cysteine substitutions can introduce ectopic disulfide bonding (typical –S–S– bond length = 2 Å), we assessed proximity to neighboring cysteines; the introduced Cys149 is > 20 Å from the nearest cysteine, making formation of a new disulfide bond unlikely under the modeled conformation (Figure S2). We next examined protein stability by computing the change in folding free energy (ΔΔG) and found that Cys149 substitution destabilizes MRI1 protein (ΔΔG = −0.74^19^ and −0.30 kcal/mol^20^).

### Arg149Cys substitution enhances ligand binding through binding-pocket compaction

Arginine is a highly polar residue, and analysis of the hydropathy index (a measure of interaction between amino acid sidechains and polar solvents) showed a localized increase in hydrophobic character at residue 149 upon substitution with Cysteine (Figure 2G). This higher hydrophobicity was confined to amino acids within the upper (residues 145→148) and the lower (residues 150→153) turns of the α-helix, indicating a localized reduction in solvent affinity, accompanied by decreased solvent accessibility (69%, Cys149 vs. 75%, Arg149).^19^ This prompted us to examine whether the Arg149Cys substitution also influences ligand-binding.

**Figure 3:**
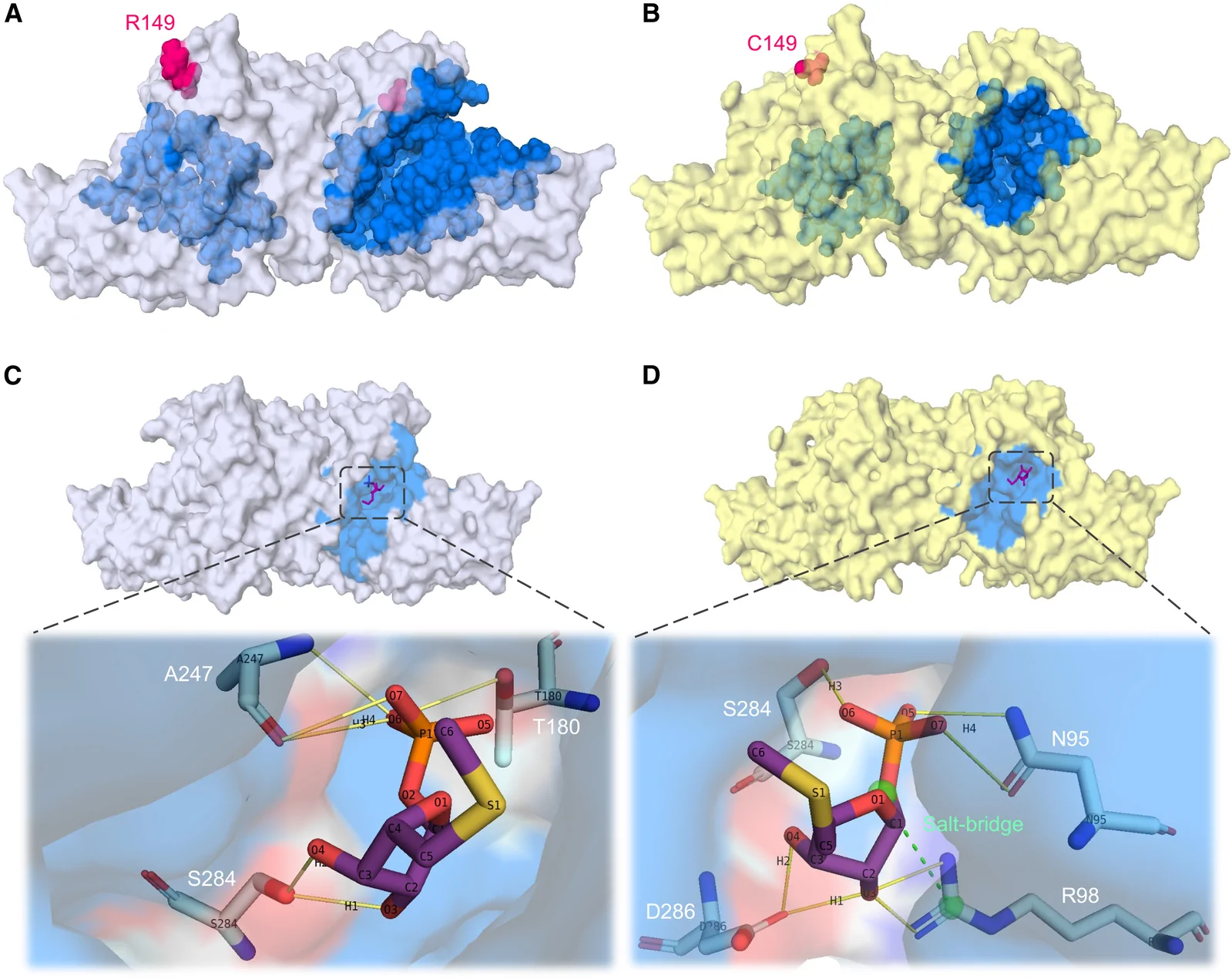
Structural and functional consequences of Arg149Cys substitution (A–D) Molecular surface representations of the MRI1 proteins—Arg149 (A) and Cys149 (B), showing the predicted ligand-binding pockets (colored in blue) in each monomer as identified by using the CASTpFold server. Average pocket size (surface area) is 1143 Å^2^ for Arg149 and 378.5 Å^2^ for Cys149 protein. Local structural views showing blind molecular docking of the MRI1 substrate (ligand), methylthioribose-1-phosphate, to the Arg149 (C) and Cys149 (D) proteins, performed using AutoDock Vina.^22^ Arg149Cys substitution improved ligand binding strengthened by the formation of a stabilizing salt bridge (green dashed line) in Cys149 protein. Amino acid residues interacting with the ligand are indicated and hydrogen bonds are shown as solid lines.

Using the CASTpFold server,^21^ we detected substantially smaller ligand-binding pockets in Cys149 protein compared with the native Arg149 protein (Figures 3A and 3B). In the Cys149 protein, each monomer contained a pocket comprising 38 residues with a surface area of ∼440 Å^2^ and a volume of ∼317 Å^3^, whereas the Arg149 protein exhibited larger pockets spanning 58 and 76 residues with surface areas of 907.1 and 1378.9 Å^2^ and volumes of 679.9 and 1407.4 Å^3^, respectively. Thirty residues were common among the identified pockets, indicating a conserved core binding region across both proteins (Figure S3). Consistent with these observations, inter-residue distance measurements within the pocket revealed reduced spatial dispersion of pocket-lining residues in Cys149 protein (data not shown), supporting a more compact binding-site geometry relative to Arg149 protein.

To understand how the smaller ligand-binding pockets influence ligand engagement, we performed blind molecular docking of the MRI1 substrate, methylthioribose-1-phosphate (MTR-1-P), to both proteins employing AutoDock Vina.^22^ Docking analyses indicated that the MTR-1-P binding site is preserved in both proteins; however, the Cys149 protein exhibited improved predicted ligand binding (interaction energy = −6.1 kcal/mol) relative to the Arg149 protein (interaction energy = −5.8 kcal/mol). In the Cys149 protein, the ligand adopts a distinct binding pose that enables formation of a stabilizing salt bridge with residue Arg98 (Figure 3C), an interaction not observed in the Arg149 complex, potentially resulting in a more favorable electrostatic stabilization of the enzyme-substrate complex and enhanced catalytic activity.

To validate our docking analysis, we tested two MRI1 variants previously shown to abolish catalytic activity (Cys168Ser and Asp248Ala)^23^; both displayed reduced interaction energies (−5.7 and −5.0 kcal/mol, respectively) relative to the Arg149 protein, supporting the sensitivity of the docking method used to detect ligand-binding differences associated with MRI1 Cys149 substitution.

### Cys149 variant enhances MRI1 catalytic activity

**Figure 4:**
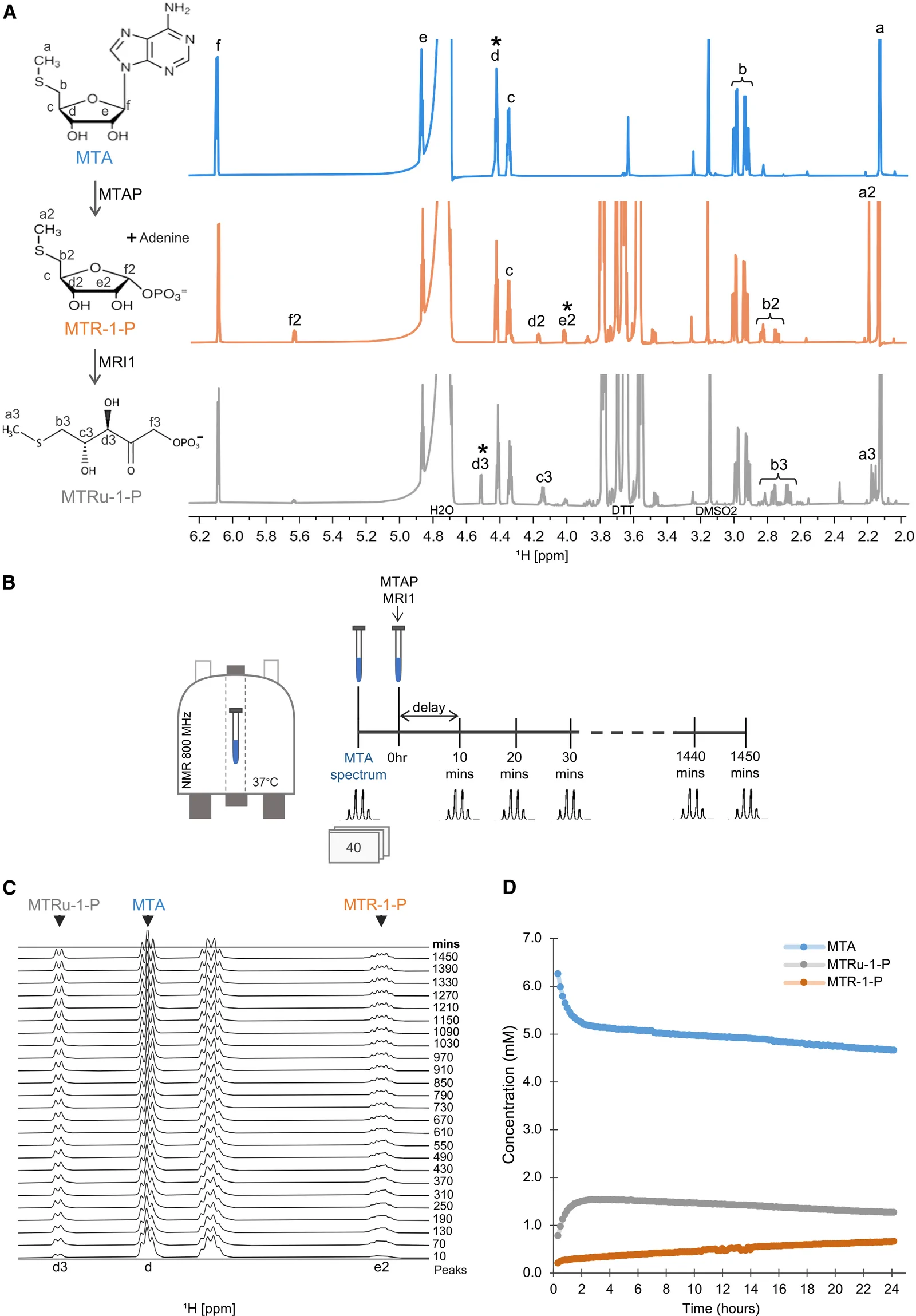
Quantitative real-time ^1^H-NMR monitoring of MRI1 catalysis (A) ^1^H-NMR spectra showing methylthioadenosine (MTA; top, blue), methylthioribose-1-phosphate (MTR-1-P; middle, orange) generated following MTAP-mediated cleavage of MTA, and methylthioribulose-1-phosphate (MTRu-1-P; bottom, gray) produced upon subsequent MRI1-catalyzed conversion. Spectra were acquired using a Bruker Avance *NEO* 800-MHz spectrometer, with 1 mM DMSO included as an internal standard. Resonance assignments for key protons are indicated and correspond to the labeled atoms in the chemical structures of the respective compounds. Peaks marked with asterisks denote distinct proton resonances used to track MTA, MTR-1-P, and MTRu-1-P formation over time. (B–D) Schematic of the quantitative real-time NMR assay workflow. Reactions were performed at 37°C in an NMR spectrometer, with sequential addition of MTAP and MRI1 followed by time-resolved acquisition of ^1^H NMR spectra to monitor substrate turnover and product formation over 24 h. Spectra were acquired in 50% [^2^H] H_2_O at 10-min intervals (5 min wait- and 5 min record-period) and each spectrum consisted of 40 scans that were signal-averaged. Time-resolved stacked ^1^H NMR spectra (C) and, corresponding concentration-time profiles (D) showing depletion of MTA (blue) and formation of MTR-1-P (orange) and MTRu-1-P (gray) over 24 h. Two independent experiments were performed (one with Arg149 and another with Cys149 MRI1 enzyme); representative spectra and concentration profiles of experiment with the native Arg149 MRI1 enzyme are shown.

The derived allele-specific differences in MRI1 ligand-binding energetics motivated us to experimentally validate the *in-silico* predictions. To examine the effects of Arg149Cys substitution on MRI1 enzyme activity, we used quantitative ^1^H-nuclear magnetic resonance (NMR) spectroscopy, which enables direct label-free tracking of reaction intermediates via proton (^1^H-atom) chemical shifts. In this study, MRI1 catalytic activity was quantified by monitoring real-time enzyme kinetics for a 2-step reaction involved in generating methionine: (1) methylthioadenosine phosphorylase (MTAP) conversion of methylthioadenosine (MTA) to MTR-1-P and (2) MRI1-mediated conversion of MTR-1-P to methylthioribulose-1-phosphate (MTRu-1-P) (Figure 4A).

Peaks for MTA were recorded in the absence of MTAP and MRI1 enzymes at ppm 2.14 (s, –CH_3_; peak a), 2.94 and 3.01 (dd, –CH_2_; peak b), 4.36 (dt, –CH; peak c), 4.43 (t, –CH; peak d), 4.88 (t, –CH; peak e), and 6.01 (d, –CH, peak f) (Figures 4A and S4); the H-atoms present in the attached adenine moiety were detected at 8.28 and 8.38 ppm (Figure S5). Following the addition of MTAP and native MRI1 Arg149 enzymes, the chemical shifts were read over a period of 24 h (Figures 4B and 4C). MRI1 substrate, MTR-1-P showed distinct resonance at ppm 2.20 (s, –CH_3_; peak a2), 2.75 and 2.83 (dd, –CH_2_; peak b2), 4.34 (m, –CH; peak c2), 4.18 (dd, –CH, peak d2), 4.02 (dd, –CH, peak e2), and 5.64 (dd, –CH, peak f2) while the released adenine displayed an upfield chemical shift relative to MTA at 8.20 and 8.25 ppm (Figure S5). MRI1 product, MTRu-1-P was characterized by well-defined peaks at ppm 2.15 (s, –CH_3_; peak a3), 2.69 and 2.78 (m, –CH_2_; peak b3), 4.16 (m, –CH; peak c3), 4.52 (d, –CH; peak d3); the peak f3 was hidden by the water peak (Figure 4A), and was only visible in a 2D spectrum (Figure S4). Distinct and non-overlapping proton resonances (indicated by asterisk) were used to uniquely track MTA (peak c), MTR-1-P (peak e2), and MTRu-1-P (peak d3) formation over time. Peak intensities from time-resolved NMR spectra collected over 24 h were converted to concentrations using the known concentration of the internal DMSO_2_ standard and plotted as a function of time (Figures 4D and S6A; Table S1). Mole balance analysis—excluding adenine co-released stoichiometrically with MTR-1-P—confirmed that MTA, MTR-1-P, and MTRu-1-P account for 94%–104% of the starting substrate concentration across the 24-h time course (Figure S6B and Table S1), confirming reaction specificity and NMR quantification accuracy. Formation of MTRu-1-P (MRI1 product) was linear during the initial 2–3 h, after which the reaction slowed, and we observed increase in MTR-1-P with time is due to reverse isomerization of MTRu-1-P back to MTR-1-P as MTRu-1-P accumulates in the absence of downstream salvage enzymes, thermodynamically favoring the reverse MRI1-catalyzed reaction as the coupled system approaches equilibrium. Therefore, subsequent kinetic analyses were performed over a shorter 4-h time window.

**Figure 5:**
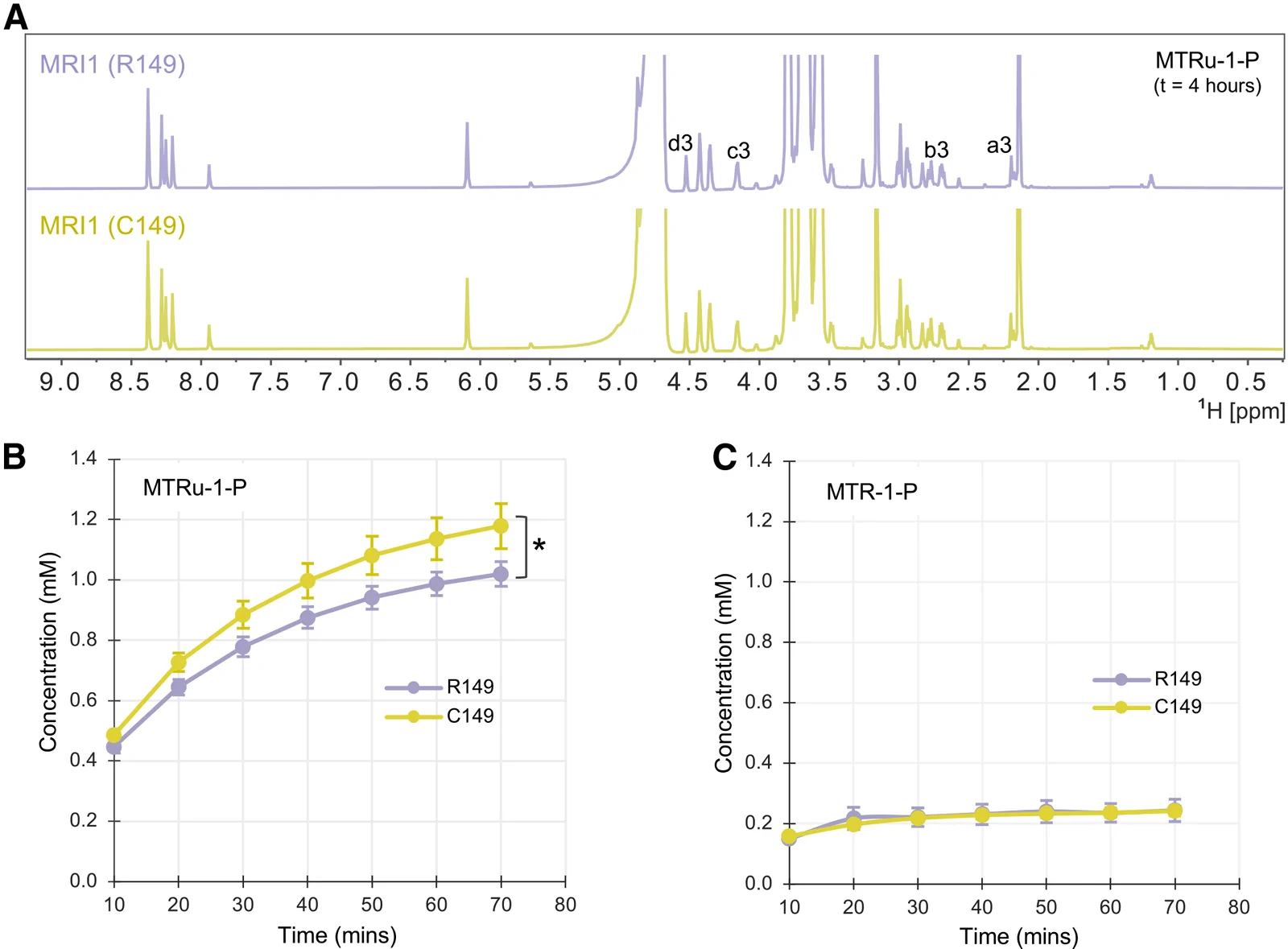
Arg149Cys enhances MRI1 catalytic activity (A) Representative ^1^H-NMR spectra of methylthioribulose-1-phosphate (MTRu-1-P, MRI1 product) at 4 h following MRI1 catalysis, comparing the Arg149 (R149, lavender) and Cys149 (C149, yellow). Key resonances of MTRu-1-P are indicated. Number of independent experiments is 4. (B) Time-dependent formation of MTRu-1-P after catalysis by MRI1 R149 (lavender) and C149 (yellow) proteins, quantified from NMR peak integration shown during the linear phase of the reaction (10–70 min). Data are plotted as mean ± standard error from four independent experiments, showing a significant increase in MTRu-1-P formation observed for the C149 protein (average increase in turnover = 27.1 nM/s; ∗*p* = 0.016, two-tailed, Wilcoxon matched-pairs signed-rank test). (C) Time-course of MTR-1-P (MRI1 substrate) levels during the reaction, indicating comparable substrate availability for Arg149 and Cys149 MRI1 proteins (*p* = 0.297, two-tailed, Wilcoxon matched-pairs signed-rank test). Number of independent experiments is 4.

To test the impact of Arg149Cys substitution on MRI1 catalytic function, we generated a recombinant human MRI1 protein harboring Cys149 and performed parallel kinetic analyses. MRI1 Cys149 enzyme retained catalytic activity (Figure 5A) and exhibited a higher rate of MTR-1-P to MTRu-1-P conversion compared with the Arg149 enzyme, with a 27.1 nM/s average increase in turnover (173.16 nM/s for Cys149 enzyme vs. 146.06 nM/s for Arg149 enzyme; *p* = 0.016, two-tailed Wilcoxon matched-pairs signed-rank test, 4 independent experiments, Figure 5B and Table S2). MTR-1-P availability (MRI1 substrate) was unchanged across assay conditions (*p* = 0.297, two-tailed Wilcoxon matched-pairs signed rank test, four independent experiments), indicating that the increased product formation reflects differences in MRI1 catalytic activity rather than substrate supply (Figure 5C). This increase in enzymatic activity due to the Arg149Cys substitution represents a gain-of-function without changes in enzyme levels.

## Discussion

In this study, we report a previously unrecognized role for a key methionine salvage enzyme, MRI1, in T2D supported by convergent genetic, biophysical, and enzymatic evidence implicating the protective Arg149Cys variant. Substitution of arginine with cysteine resulted in a compact ligand-binding pocket enabling better substrate engagement and improved MRI1 catalytic efficiency. Counterintuitively, the Arg149Cys allele which is protective for T2D is enriched in a population isolate that has high T2D disease burden, suggesting that high-risk populations provide a powerful setting to detect protective variants that may only be evident against a background of chronic metabolic stress.^9^^,^^24^ MRI1 Arg149Cys variant appears to function in this manner, enhancing methionine salvage efficiency and delaying disease onset without altering overall disease burden at the population level. Although this protective substitution is extremely rare in the general population, this study reveals a pathway-level mechanism by which methionine salvage efficiency can modify T2D risk.

Methionine is an essential amino acid obtained exclusively from diet and is a critical substrate for numerous key biological processes, including protein synthesis, methylation reactions, polyamine biosynthesis, redox homeostasis, and folate metabolism.^25^ The universally conserved methionine salvage pathway restores methionine from *S*-adenosyl-methionine (SAM), which serves as a methyl donor for all methylation reactions.^25^^,^^26^ We observed that the Cys149 MRI1 enzyme catalyzed the conversion of MTR-1-P to MTRu-1-P at a faster rate than the Arg149 enzyme. MTRu-1-P is further metabolized to methylthio-2-oxobutanoate which is then converted to methionine.^26^ Because these downstream steps of the methionine salvage pathway are irreversible, increased flux through the MRI1-catalyzed step is expected to propagate through the pathway, resulting in greater net regeneration of methionine. Methionine metabolism is dysregulated across a broad spectrum of conditions including T2D,^27^ non-alcoholic fatty liver disease,^28^ cardiovascular disease,^29^ neurodegeneration,^30^ and aging^31^—all characterized by sustained demand on SAM-dependent methylation, redox homeostasis, and biosynthetic capacity. Our identification of MRI1 as a functionally tunable enzyme whose activity can be meaningfully enhanced by a single amino acid substitution suggests that MRI1 activation may have therapeutic utility beyond T2D, in conditions where intracellular methionine demand chronically exceeds recycling capacity.

We observed a positive correlation between *MRI1* and *INS* expression in human islets, suggesting that methionine capacity increases in parallel with insulin production. Higher plasma methionine levels correlated with lower insulin sensitivity (i.e., higher insulin resistance) in our study, consistent with a prior report of a positive correlation between serum methionine levels and insulin resistance (HOMA-IR, Spearman’s r = 0.16, *p* < 0.002, *n* = 560),^32^ collectively suggesting that methionine metabolism is relevant to insulin-resistant state. During early systemic insulin resistance state, islet β-cells experience sustained biosynthetic stress to increase insulin production to maintain glucose homeostasis,^33^^,^^34^ requiring increased protein synthesis, methylation reactions, and maintenance of redox homeostasis—processes that are critically dependent on methionine.^25^ Thus, enhanced MRI1 activity might buffer β-cells against metabolic stress imposed by insulin resistance, contributing to preserved insulin production and delayed disease progression. Small molecule MRI1 activators mimicking Cys149 conformation could serve as β-cell protective agents buffering against the biosynthetic, methylation, and redox stresses that drive progressive β-cell failure—a therapeutic approach distinct from existing T2D therapeutics, none of which target methionine metabolism.

Beyond islets, *MRI1* is expressed at similar levels in key insulin-responsive tissues-adipose, muscle, and liver. We speculate that MRI1 functions as a systemic regulator of insulin sensitivity across tissues. For instance, in human adipose tissue, *MRI1* expression is negatively correlated with circulating insulin, circulating glucose, HOMA-IR, and adipocyte volume (all r > −0.10, FDR-adjusted *p* < 0.01, *n* = 15 [except adipocyte volume: *n* = 6]).^35^ This implies that higher *MRI1* expression reflects a metabolically healthier adipose phenotype characterized by improved insulin sensitivity, reduced adipocyte hypertrophy, and better systemic glucose homeostasis. Comparable clinical data for *MRI1* expression in muscle and liver are not currently available; therefore, its relationship with metabolic parameters in these tissues cannot be assessed. MRI1 is not only tissue-expressed but also present at measurable circulating levels (13 ng/L)^36^ in human blood plasma.

Intriguingly, we show that the Arg149Cys substitution reduces protein stability yet increases catalytic activity—an apparent paradox that is in fact a well-established phenomenon in enzymology formally described as the “stability-activity trade-off”^37^; and the MRI1 Cys149 variant is a compelling example of this principle. The spatial context of Arg149Cys supports this paradox—the substitution resides distal to the MRI1 active site. A recent study demonstrated that while active-site mutations create preorganized catalytic geometries, the distal mutations enhance catalysis specifically by facilitating substrate binding and product release through tuning structural dynamics.^38^ Our data mirrors this dynamic precisely—the distal Arg149Cys substitution promotes binding pocket compaction, improved substrate engagement, and formation of a new stabilizing Arg98 salt bridge, collectively explaining how a structurally peripheral change simultaneously introduces mild destabilization while delivering a meaningful and measurable gain in catalytic efficiency.

The methionine salvage pathway is an established pharmacological space—the MAT2A inhibitor AG-270 has entered phase I clinical trials for MTAP-deleted cancers,^39^ demonstrating that enzymes in this pathway are tractable drug targets. The structural mechanism we identify—binding pocket compaction enabling improved substrate engagement—provides a rational basis for structure-guided design of small molecule MRI1 activators, for which our generated Cys149 homology model serves as a ready-made structural template. Unlike inhibitor-based strategies, activating a physiological recycling enzyme carries lower on-target toxicity risk since the intervention enhances rather than disrupts a normal cellular process.

Notably, in this study, we provide high-resolution, well-resolved ^1^H-NMR spectra for MTA, MTR-1-P, and MTRu-1-P, which will serve as a valuable reference for future studies of the methionine salvage pathway. Although earlier reports have described NMR spectra of MTRu-1-P,^23^^,^^40^^,^^41^ these studies are relatively dated and were limited by lower spectral resolution and incomplete resonance assignments. Our study addresses these limitations by providing clearly assigned, high-quality spectra suitable for quantitative and comparative analyses.

In summary, we report methionine salvage efficiency as previously unappreciated modifier of T2D risk and characterize MRI1 Arg149Cys substitution. Our findings support a model in which increased intracellular methionine recycling buffers metabolic tissues against the biosynthetic, epigenetic, and redox stresses imposed by insulin resistance, contributing to delayed disease onset and underscores the value of leveraging population diversity to uncover endogenous mechanisms of metabolic resilience. In addition, this study uniquely illustrates how quantitative NMR can be applied to directly and mechanistically evaluate the functional consequences of coding genetic variants, enabling real-time measurement of enzymatic flux and linking human genetic variation to downstream molecular activity without reliance on indirect or coupled assays.

### Limitations of the study

While our findings provide valuable insights, some limitations should be considered when interpreting the results of the study. Differences in predicted ligand-binding energies of MRI1 Arg149 and Cys149 enzymes were modest and provide only an approximate estimate of binding affinities. While molecular dynamics simulations would have added valuable computational depth to the structural analysis, we deliberately prioritized direct experimental validation of the structural predictions over additional computational modeling. We chose to acquire NMR spectra in 50% D_2_O/50% H_2_O to prioritize physiological relevance over conventional structural NMR conditions, a choice that is both a strength and a limitation of the study. The mixed solvent system provides conditions that are more representative of the biological environment in which MTAP and MRI1 functions; however, differences in solvent composition may influence hydrogen-bonding interactions and protonation equilibria.^42^ Subtle variation in the chemical shifts of hydroxyl- and phosphate-proximal protons relative to prior studies^43^^,^^44^ acquired in 100% D_2_O may in part reflect differences in solvent composition, among other possible contributing factors, and should be considered when making direct spectral comparisons across studies. Further, MRI1 catalytic activity was evaluated in a simplified two-enzyme system that may not fully capture regulatory influences and protein–protein interactions that may influence MRI1 activity in cells. Although the Arg149Cys substitution enhances MRI1 catalytic activity *in vitro*, the physiological link between improved methionine regeneration and systemic insulin sensitivity remains indirect, as methionine salvage pathway flux and downstream metabolic consequences were not measured *in vivo*. We attempted to quantify methionine levels in cell lysate and culture media of transfected HepG2 cells as a direct readout of enhanced MRI1 activity following overexpression of Arg149 and Cys149 constructs using a fluorometric assay (data not shown); however, this was unsuccessful as free intracellular methionine is maintained at very low concentrations due to its rapid consumption across multiple metabolic pathways, making steady-state methionine levels an insensitive proxy for salvage flux. The definitive approach to directly measure salvage flux in intact cells would be ^13^C-MTA isotope tracing with LC-MS/MS in which cells are pulsed with isotopically labeled ^13^C-MTA as the sole salvage substrate, and the subsequent incorporation of ^13^C label into successive pathway intermediates (MTR-1-P and MTRu-1-P) and ultimately into ^13^C-methionine is tracked by liquid chromatography-tandem mass spectrometry. However, this methodology requires specialized isotope-labeling infrastructure and LC-MS/MS instrumentation that were not available within the scope and timeline of the current study. Addressing these questions through metabolomic, cellular, and *in vivo* studies will help clarify the physiological role of MRI1 in metabolic regulation and its potential contribution to protection against T2D.

## Resource availability

### Lead contact

Further information and requests for resources should be directed to the lead contact, Leslie J. Baier (lbaier@phx.niddk.nih.gov).

### Materials availability

The human MRI1 proteins (MRI1_Arg149 and MRI1_Cys149) generated in this study are available from the lead contact upon reasonable request.

### Data and code availability

Data on *MRI1* expression across human tissues, its correlation with *INS* expression, and its relative expression compared with other islet-expressed genes were obtained from the Islet Gene View (https://mae.crc.med.lu.se/IsletGeneView/). MRI1 RNA and protein expression in pancreatic islets from individuals with T2D and without diabetes (ND) were retrieved from HumanIslets.com (https://www.humanislets.com/#/). The computational tools used for the *in-silico* analyses are described throughout the manuscript. ^1^H-NMR chemical shifts assignments of MTA, MTR-1-P, and MTRu-1-P have been deposited in the Biological Magnetic Resonance Databank (BMRbig, entry id: bmrbig141, https://doi.org/10.13018/bmrbig141, https://bmrbig.bmrb.io/released/bmrbig141).

## Acknowledgments

This research was supported (in part) by the 10.13039/100030692Intramural Research Program of the 10.13039/100000062National Institute of Diabetes and Digestive and Kidney Diseases (10.13039/100000062NIDDK) within the 10.13039/100000002National Institutes of Health (10.13039/100000002NIH). The contributions of the NIH author(s) are considered Works of the United States Government. The findings and conclusions presented in this paper are those of the author(s) and do not necessarily reflect the views of the 10.13039/100000002NIH or the U.S. 10.13039/100000016Department of Health and Human Services. The authors thank Dr. Vlad Kumirov (Department of Chemistry and Biochemistry, College of Science, University of Arizona, Tucson, USA) for his valuable assistance with NMR standardization and data acquisition.

## Author contributions

Conceptualization, K.B., M.T., and L.J.B.; methodology, K.B. and M.T.; investigation, K.B. and K.F.; formal analysis, K.B. and P.P.; writing – original draft, K.B.; writing – review and editing, M.T., P.P., and L.J.B.; supervision, L.J.B. and M.T.; resources, L.J.B.; funding acquisition, L.J.B.

## Declaration of interests

The authors have no relevant financial or non-financial interests to disclose.

## STAR★Methods

### Key resources table

**Table:** 

REAGENT or RESOURCE	SOURCE	IDENTIFIER
**Chemicals, peptides, and recombinant proteins**		

MTAP enzyme	Origene	Cat# TP306024
MRI1 Arg149 enzyme (custom-made)	Genscript	https://www.genscript.com/recombinant-protein-services.html
MRI1 Cys149 enzyme (custom-made)	Genscript	https://www.genscript.com/recombinant-protein-services.html
MTA	Sigma-Aldrich	Cat#260585
Dimethyl sulphone	Sigma-Aldrich	Cat#41867
D2O	Sigma-Aldrich	Cat#1133660009

**Deposited data**		

^1^H-NMR chemical shifts assignments of MTA, MTR-1-P and MTRu-1-P	Current study	Entry id: bmrbig141 (https://doi.org/10.13018/bmrbig141, https://bmrbig.bmrb.io/released/bmrbig141)

**Software and algorithms**		

PyMOL	Schrödinger	https://www.pymol.org/
Site Directed Mutator (SDM)	University of Cambridge	https://compbio.medschl.cam.ac.uk/sdm2/
Cologne University Protein Stability Analysis Tool (CUPSAT)	BRENDA	https://cupsat.brenda-enzymes.org/
ProtScale (ExPASy)	Swiss Institute of Bioinformatics	https://web.expasy.org/protscale/
GalaxyWEB	Seoul National University	https://galaxy.seoklab.org/
CB-Dock	Sichuan University	https://cadd.labshare.cn/cb-dock3/php/index_v3.php

### Method details

#### Bioinformatic and biophysical analyses

Multiple sequence alignments of MRI1 across vertebrate species were generated using Multiz, and evolutionary conservation was assessed using PhastCons and PhyloP scores from the PHAST package via the UCSC Genome Browser.^17^ Conserved protein domains within MRI1 isoforms were identified using the NCBI Conserved Domain Database.^18^ Protein structures were visualized using PyMOL (The PyMOL Molecular Graphics System, Version 3.0 Schrödinger, LLC.). Effect of Arg149Cys substitution on MRI1 protein stability was evaluated using Site Directed Mutator (SDM)^19^ and Cologne University Protein Stability Analysis Tool (CUPSAT).^20^ Hydrophobicity profiles were generated using the Kyte–Doolittle method^45^ implemented in ProtScale (ExPASy)^46^ with the default window size.

#### Referred databases

Data on *MRI1* expression across human tissues, its correlation with *INS* expression, and its relative expression compared with other islet-expressed genes were sourced from the Islet Gene View^14^ (https://mae.crc.med.lu.se/IsletGeneView/). MRI1 RNA and protein expression in pancreatic islets from individuals with T2D and without diabetes (ND) were retrieved from HumanIslets.com^15^ (https://www.humanislets.com/#/). MRI1 expression in adipose tissue and its correlations with metabolic parameters were obtained from the Adipose Tissue Knowledge Portal^35^ (https://adiposetissue.org/summary?gene=MRI1). Circulatory levels of MRI1 were reported from Human Protein Atlas^36^ (https://www.proteinatlas.org/ENSG00000037757-MRI1/blood).

#### Structure prediction of MRI1 Cys149 protein

Fully resolved X-ray crystal structure of native human MRI1 protein (Arg149) was obtained from the Protein Data Bank (PDB ID: 4LDQ) and used as a template to generate the structure of MRI1 Cys149 protein using the GalaxyWEB^47^ protein structure prediction server (https://galaxy.seoklab.org/). Template-based (homology) modeling was performed employing the GalaxyTBM pipeline.^48^ MRI1 protein sequence with the substituted amino acid was submitted to GalaxyTBM with default parameters. GalaxyTBM identifies homologous template structures from PDB, constructs an initial model using single or multiple templates, and refines locally unreliable regions through targeted rebuilding. Generated models were ranked by the server’s internal scoring function, and the top-ranked model was selected for subsequent structural analyses.

#### Molecular docking

Blind molecular docking was performed using the CB-Dock web server,^49^ which employs AutoDock Vina^22^ for docking simulations. MRI1 Arg149 and Cys149 protein structures were docked independently using default parameters, allowing automatic detection of binding cavities across the entire protein surface. Docking poses were ranked by the AutoDock Vina binding affinity score, and the lowest interaction energy was used to compare docking scores between the WT and mutant structures.

#### Proteins used in enzymatic assays

Human MTAP protein (NP_002442) was purchased from Origene (Rockville, MD, USA). Human MRI1 Arg149 (NP_001026897) and Cys149 proteins were custom synthesized by Genscript (Piscataway, NJ, USA) and were stored in a buffer containing 50 mM Tris-HCl, 150 mM KCl, pH 8.0. The purity of the synthesized MRI1 proteins was ensured to be ≥ 90% (SDS-PAGE) and their mass was confirmed by LC-MS.

#### Acquisition of ^1^H-NMR spectra

MRI1 substrate methylthioribose-1-phosphate (MTR-1-P) was prepared enzymatically by incubating 3 mM methylthioadenosine (MTA) with 1 μM MTAP enzyme in a buffer containing 25 mM NaPi (pH 7.4), 0.5 mM EDTA (pH 8.0), 1 μM DTT at 37°C. The experiment was conducted with/without MRI1 protein (1μM) and the conversion of MTR-1-P to methylthioribulose-1-phosphate (MTRu-1-P) was monitored by ^1^H-nuclear magnetic resonance (NMR) spectrometry using the internal standard DMSO2 at a known concentration of 1 mM. A Bruker Avance NEO 800-MHz spectrometer was used to collect the NMR data. A sample consisting of MTA and the complete reaction buffer without the MTAP and MRI1 enzymes was first run on its own. This 0-hr spectrum was used to set up the NMR parameters (the water signal was suppressed with pre-saturation) and identify the peaks corresponding to buffer components, residual water signal near 4.7 ppm (parts per million of the applied magnetic field) and the internal standard DMSO2 peak at 3.16 ppm. Besides a 1D spectra at 0-hour, 2D NMR spectra (^13^C-heteronuclear single quantum correlation, HSQC, and heteronuclear multiple bond correlation, HMBC) were taken to identify distinct resonances (peaks) corresponding to each compound in the reaction. A timer was set at the beginning of the addition of MTAP and MRI1 enzymes to account for the delay time (the time before the collection of the first NMR spectra after enzymes were added); a delay time of 10 mins was included in the calculations. The final reaction volume was 500μl and the reaction temperature was 37°C. The spectra were obtained in 50% [^2^H] H_2_O after every 10 mins (5 mins wait- and 5 mins record-period). The initial experiment was monitored for 24 hours (144 spectra per experiment), and the subsequent experiments were followed over a reaction time of 4 hours (25 spectra per experiment). Each spectrum consisted of 40 scans that were signal-averaged (each scan had an acquisition time of 2.23 seconds, along with 5 seconds relaxation delay between scans). At 250 mins (end of a 4-hour experiment), a 1D and 2D spectra were taken after re-shimming the magnet to check the broadness of the peaks and identify peaks corresponding to MTR-1-P and MTRu-1-P, respectively.

#### NMR data quantification and kinetic analysis

At each spectrum in the kinetics array, peak intensity of a compound (MTA or MTR-1-P or MTRu-1-P) was divided by the peak intensity of DMSO2 to obtain concentration and correct for any time-dependent changes in the magnetic field. The concentrations were plotted against time, and the rate of the reaction was calculated from the slope of the graph when the reaction was linear assuming a first order kinetics (timepoints considered: 10, 20, 30, 40, 50, 60 and 70 minutes). Linear regression yielded trendline fits with coefficient of determination (r^2^) greater than 0.90 for all individual independent experiments.

### Quantification and statistical analysis

Details of all data comparisons including sample size (n), sample type, statistical tests, and measures of center and dispersion are provided in the corresponding figure legends. For data sourced from the referenced databases, r- and *p*-values are presented as originally reported by the respective databases. Comparison of the rates of MTR-1-P and MTRu-1-P formation catalyzed by the MRI1 Arg149 and Cys149 proteins were analyzed using a two-tailed Wilcoxon matched-pairs signed-rank test in GraphPad Prism, with concentrations compared at matched time points.

## Supplemental information

Document S1. Figures S1–S6

Table S1. Concentration-time data for MTA, MTR-1-P, and MTRu-1-P across the 24-h real-time ¹H-NMR kinetic assay for MRI1 Arg149 and Cys149

Table S2. Kinetics comparison of MTR-1-P and MTRu-1-P concentrations for MRI1 Arg149 and Cys149 enzymes
