# Supplementary material for: A functional missense variant in MRI1 links methionine salvage to reduced type 2 diabetes risk
Source: iScience. 2026 Aug 20;29(9):117243. doi: 10.1016/j.isci.2026.117243 (PMC13524455; doi:10.1016/j.isci.2026.117243)
Supplement: Supplementary file 1 [file mmc1.pdf]

iScience, Volume *29*

Supplemental information

A functional missense variant in *MRI1* links methionine salvage to reduced type 2 diabetes risk

Khushdeep Bandesh, Michael Traurig, Kendrick Freeland, Paolo Piaggi, and Leslie J. Baier

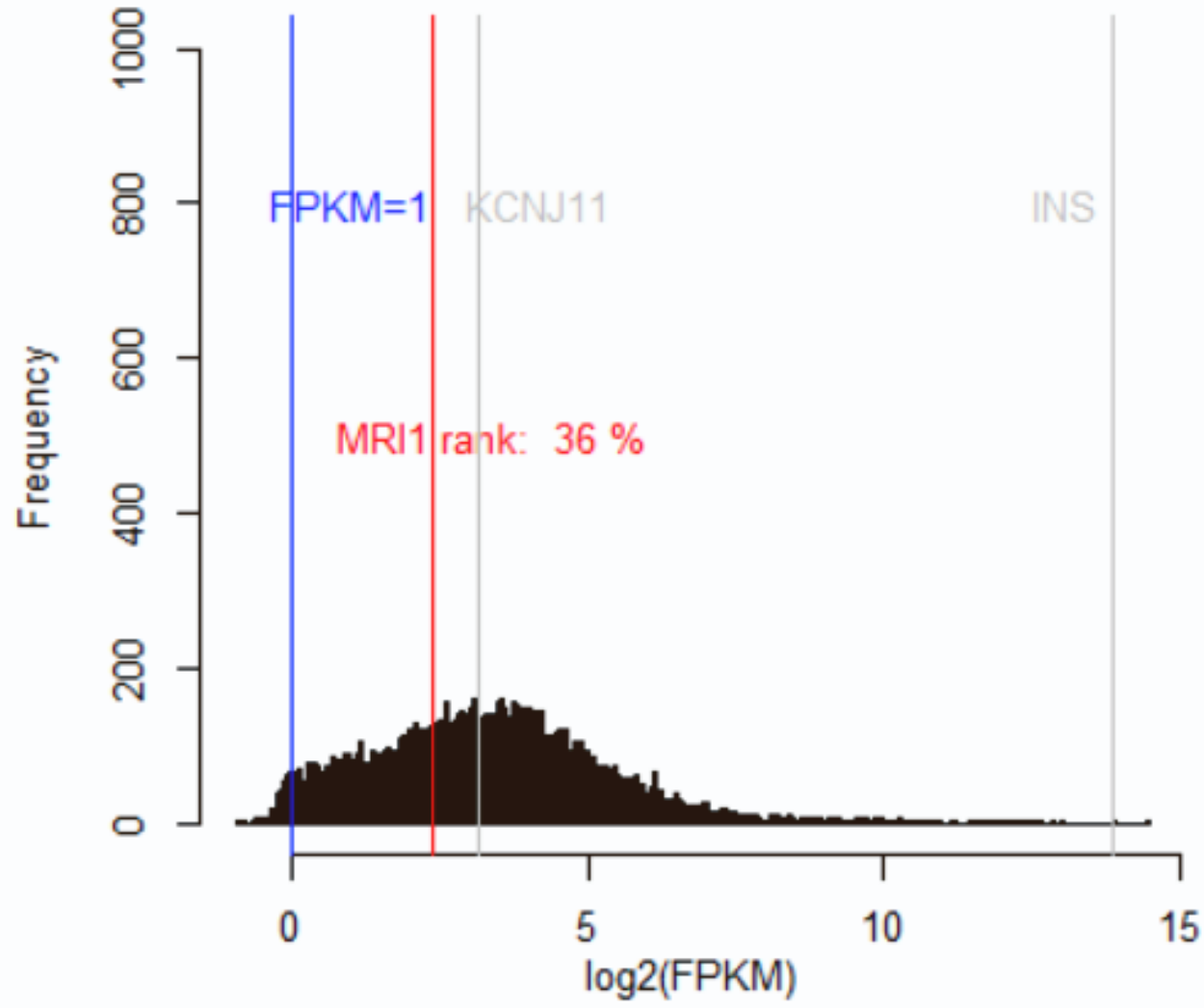

Supplementary Figure 1: MRI1 expression relative to genes expressed in human pancreatic islets. Histogram showing the distribution of log_2-transformed FPKM expression values for all genes expressed in human pancreatic islets (Islet Gene View) [PMID: 35948367]. The position of MRI1 is indicated by the red vertical line, corresponding to the 36th percentile of islet-expressed genes. The blue vertical line denotes the expression threshold of FPKM = 1. Representative islet-expressed genes, including *KCNJ11* and *INS*, are indicated for reference.

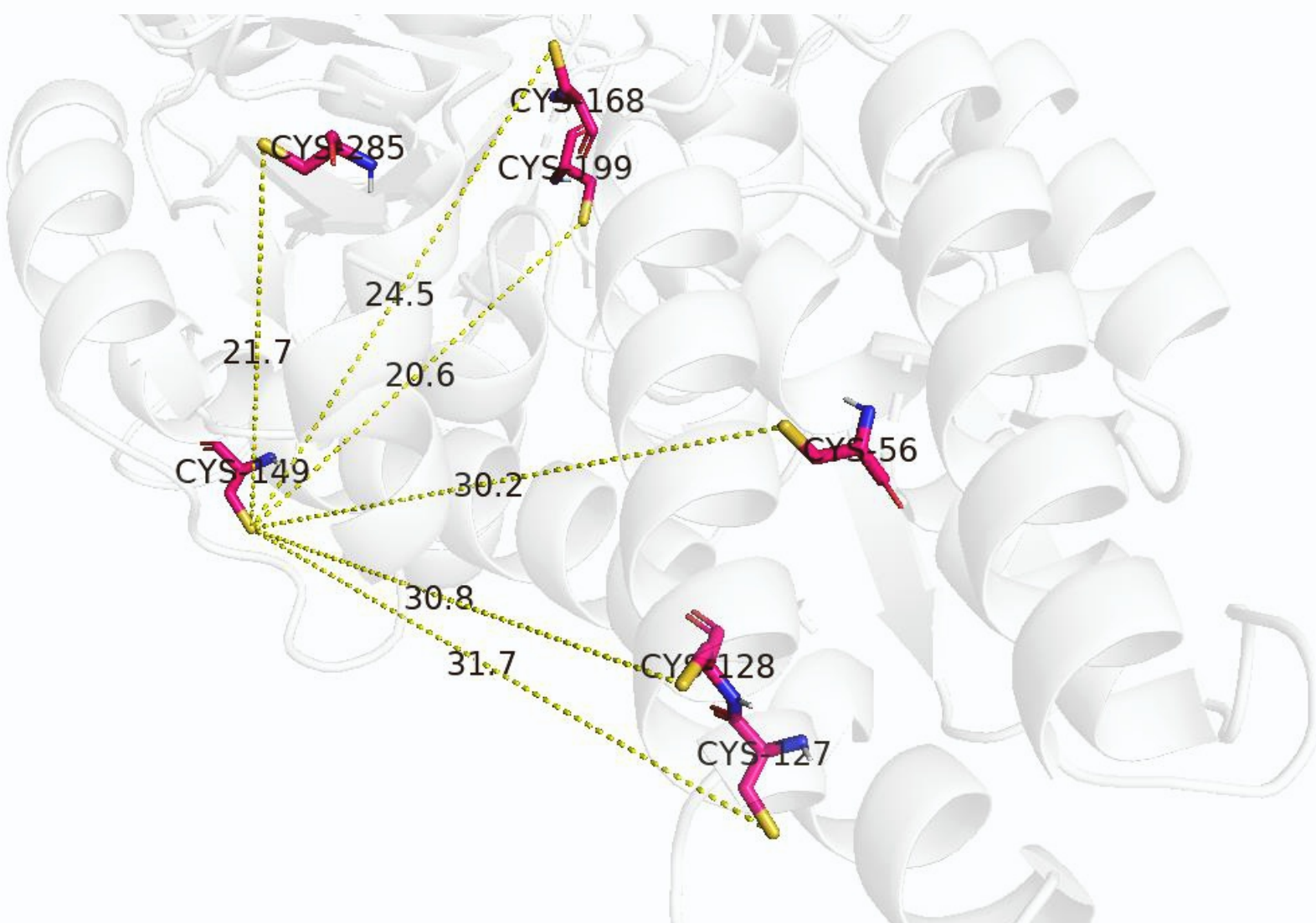

Supplementary Figure 2: Disulfide bond potential of the substituted Cys149 within MRI1. Spatial proximity of cysteine residues within the MRI1 protein. Ribbon representation of the folded human MRI1 protein highlighting cysteine residues shown in stick representation. Pairwise distances between S-atoms of C149 and other cysteines side chains are indicated by dashed lines (Å).

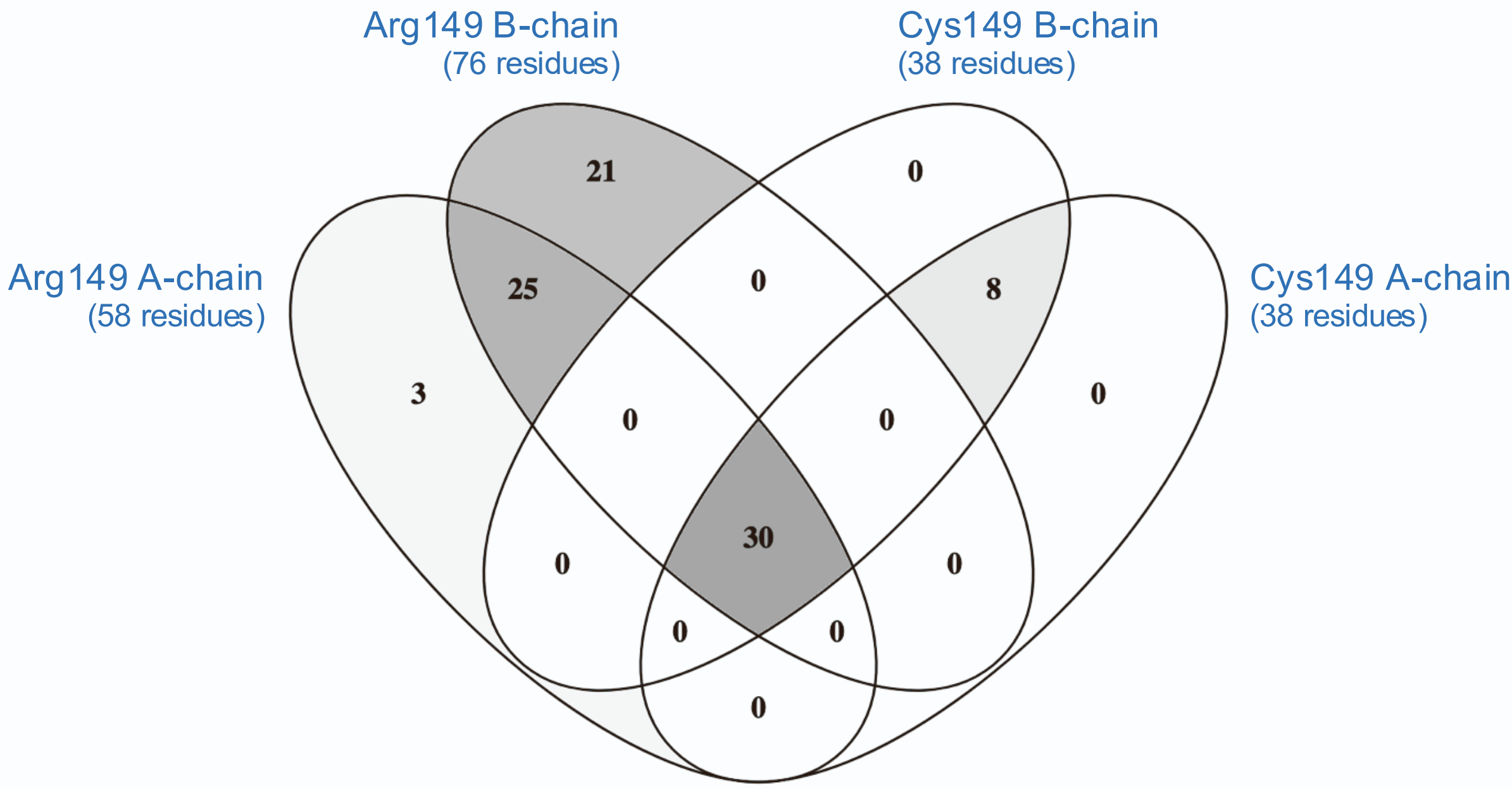

Supplementary Figure 3: Overlap of ligand-binding residues of the MRI1 Arg149 and Cys149 proteins. Venn diagram showing the overlap of residues constituting the predicted ligand-binding pockets in the A- and B-chains of the MRI1 Arg149 and Cys149 proteins. The total number of pocket-forming residues identified in each chain is indicated in parentheses.

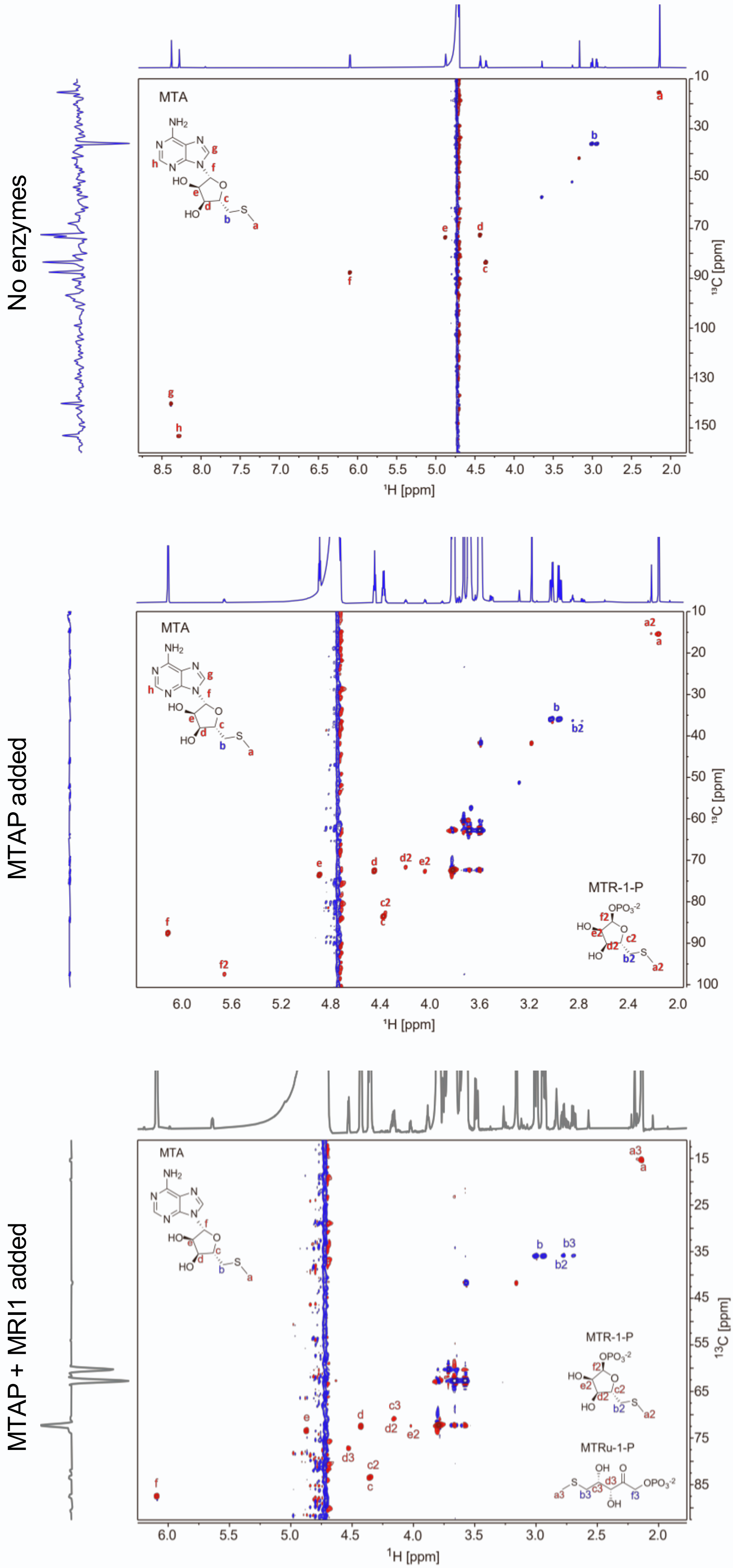

Supplementary Figure 4: 2D ^{1}H–^{13}C HSQC NMR spectra tracking the two-step MTAP/MRI1 reaction. Representative 2D ^{1}H–^{13}C NMR spectra acquired under three reaction conditions: without enzymes (top), MTAP only (middle), and MTAP + MRI1 (bottom). In the absence of enzymes, only resonances corresponding to methylthioadenosine (MTA) are observed. Addition of MTAP results in the appearance of methylthioribose-1-phosphate (MTR-1-P) resonances, whereas subsequent addition of MRI1 leads to the formation of methylthioribulose-1-phosphate (MTRu-1-P). Assigned cross-peaks are labeled and correspond to the indicated atoms in the chemical structures shown.

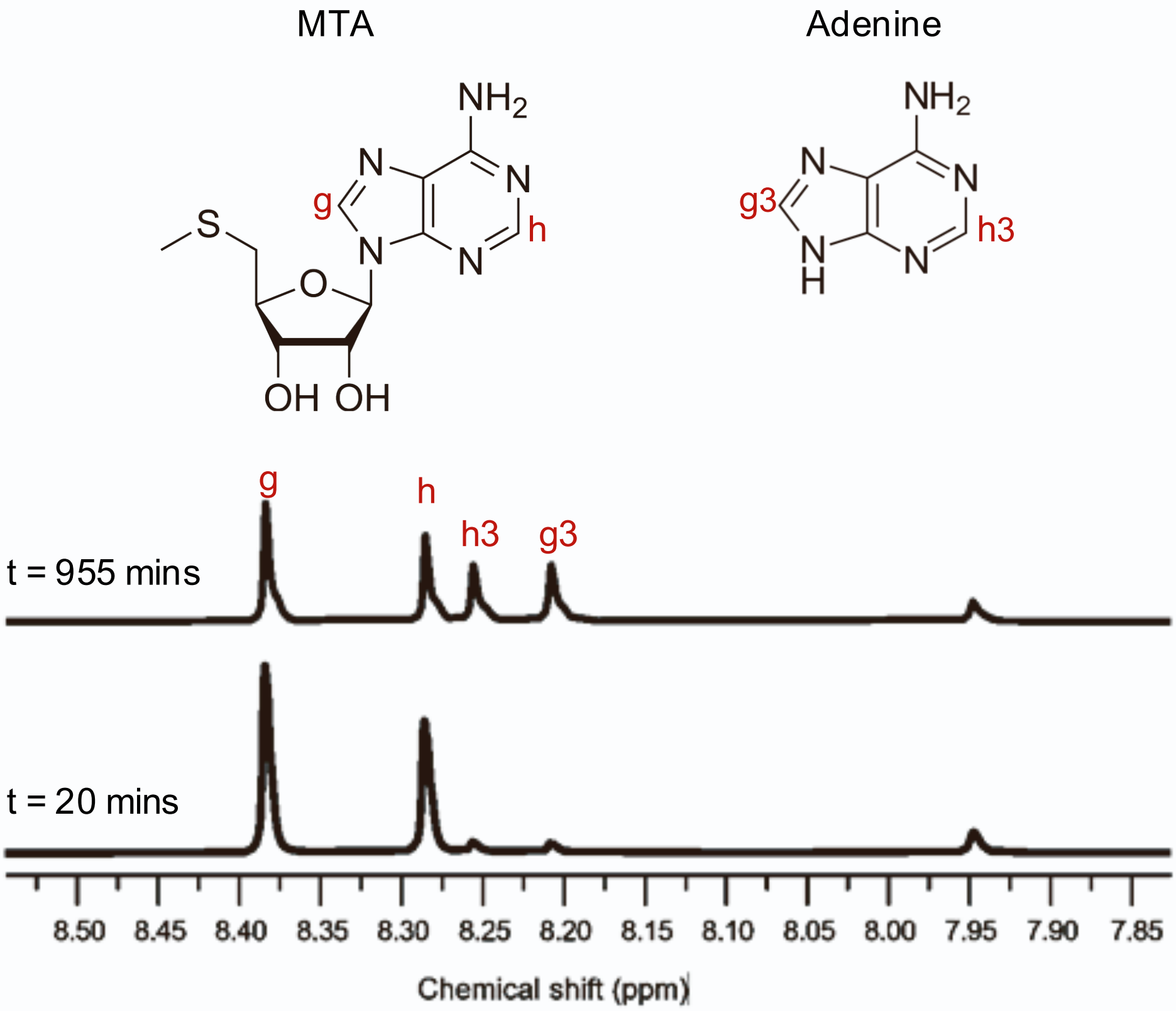

Supplementary Figure 5: Adenine accumulation over time in the MTAP/MRI1 reaction by ^{1}H-NMR. Representative ^{1}H-NMR spectra acquired at early (t = 20 min) and late (t = 955 min) time points showing the appearance and accumulation of adenine following MTAP-catalyzed cleavage of MTA. Assigned proton resonances corresponding to adenine (g3, h3) and MTA (g, h) are indicated and mapped to the labeled atoms in the chemical structures shown above the spectra. The increase in adenine-specific resonances over time confirmed MTAP-mediated release of adenine.

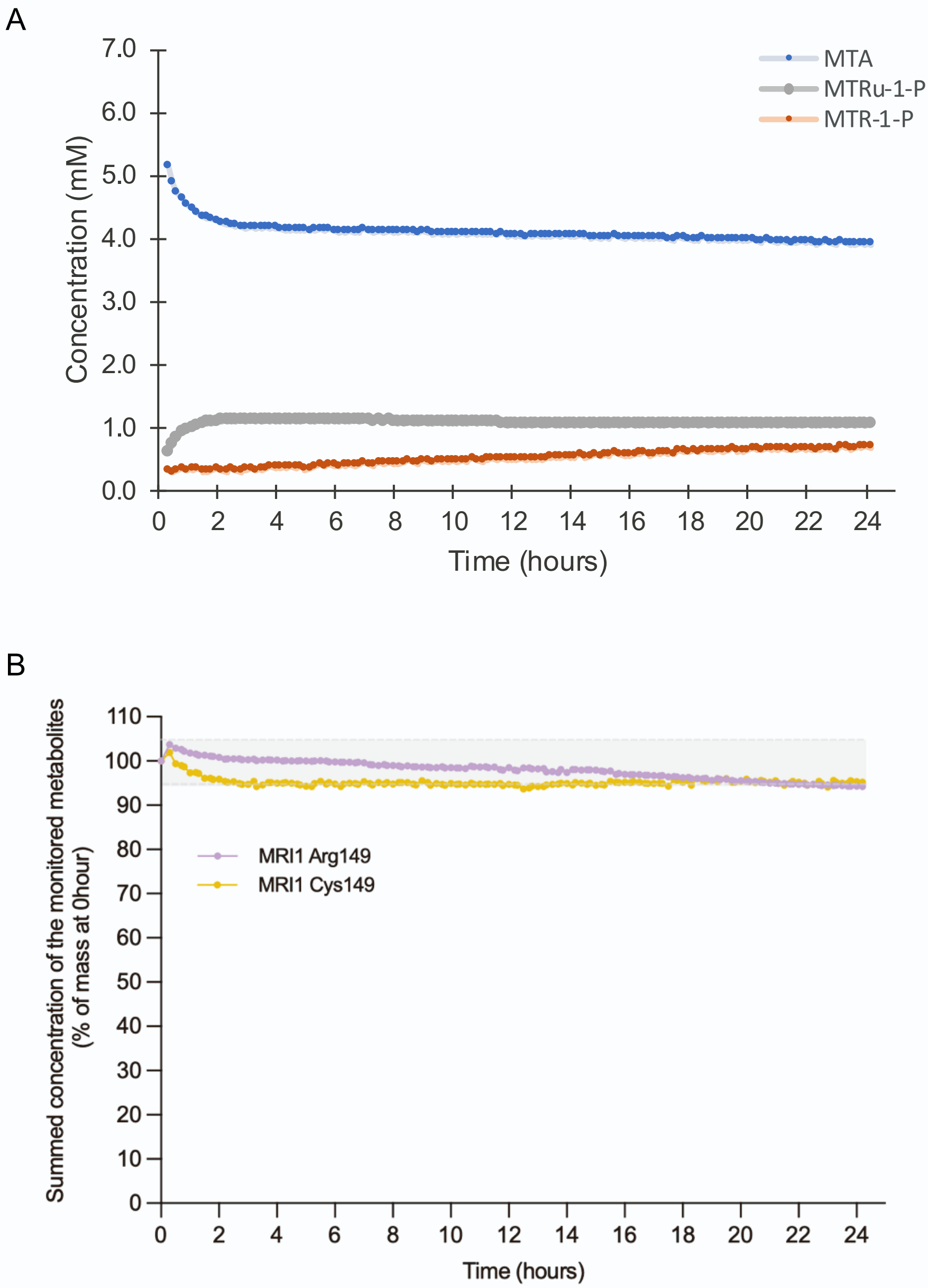

Supplementary Figure 6: Concentration-time profile of MRI1 Cys149 enzyme kinetics and mole balance analysis of the coupled MTAP/MRI1 reaction. (A) Concentration-time profiles showing depletion of MTA (blue, methylthioadenosine) and formation of MTR-1-P (orange, methylthioribose-1-phosphate) and MTRu-1-P (gray, methylthioribulose-1-phosphate) over 24 hours where the MRI1 catalyzed step was performed using MRI1 Cys149 protein. Metabolite concentrations were derived from peak integrals referenced to a known concentration of DMSO internal standard. **(B)** Mole balance analysis of the coupled MTAP/MRI1 NMR kinetic assay. Summed concentration of MTA, MTR-1-P, and MTRu-1-P expressed as a percentage of the concentration at t = 0 hours — plotted across the 24-hour time course for MRI1 Arg149 (purple) and MRI1 Cys149 (yellow) experiments. Adenine — released stoichiometrically as a co-product of the MTAP-catalyzed reaction — was not quantified and is not included in the three-species sum. The shaded gray region represents the accepted ±5% quantitative NMR error range (PMID: 18844373).
